# Diagnostic Accuracy of Cranial Ultrasound for the Detection of Intracranial Hemorrhage in Preterm Neonates Using Magnetic Resonance Imaging As the Gold Standard

**DOI:** 10.7759/cureus.73732

**Published:** 2024-11-15

**Authors:** Maryam Sheikh, Sadia Shabbir

**Affiliations:** 1 Department of Imaging and Radiology, Children's Hospital Lahore, University of Child Health Sciences, Lahore, PAK; 2 Department of Radiology, Children's Hospital Lahore, University of Child Health Sciences, Lahore, PAK

**Keywords:** cranial ultrasound, diagnostic accuracy, intracranial hemorrhage (ich), magnetic resonance imaging, preterm neonates

## Abstract

Background and objective

Intracranial hemorrhage (ICH) and white-matter damage are the main brain injuries in preterm infants. Magnetic resonance imaging (MRI) is the best way to examine cerebral bleeding. The evidence on cranial ultrasound diagnostic accuracy in neonates is limited in Pakistani publications, which show variability in evidence, necessitating the present study. The aim is to find out the diagnostic accuracy of cranial ultrasound for detecting ICH in preterm neonates, taking MRI as the gold standard.

Methodology

A cross-sectional study was carried out in the Department of Radiology, The Children’s Hospital, Lahore, Pakistan, from June 19, 2018 to December 18, 2018. The study involved 103 preterm neonates of both genders aged between 1 and 27 days and presenting with suspicion of ICH. The cranial ultrasound with MRI (gold standard) was used to diagnose ICH, and the results of the cranial ultrasound were assessed accordingly. The diagnostic performance of ultrasound was estimated using parameters, e.g., sensitivity, specificity, accuracy, and positive and negative predictive values. The p < 0.05 was taken as statistically significant.

Results

The neonates had a mean age of 9.9 ± 6.6 days. There were 53 (51.5%) male and 50 (48.5%) female neonates. The cranial ultrasound diagnosed ICH in 39 (37.9%) neonates, which is confirmed by MRI as the gold standard. Cranial ultrasound had 100.0% sensitivity, 90.62% specificity, 94.17% accuracy, 86.67% positive predictive value, and 100.0% negative predictive value for detecting ICH in preterm neonates. Similar diagnostic performance was seen across age and gender groups (p > 0.05).

Conclusion

Cranial ultrasound was 100% sensitive, 90.6% specific, and 94.2% accurate in identifying cerebral bleeding in preterm neonates. The noninvasive, ionizing radiation-free nature and availability at the bedside of cranial ultrasound make it a promising future modality.

## Introduction

The main brain injuries in preterm infants are intracranial hemorrhage (ICH) and white matter damage [1]. Sub-dural hemorrhage, germinal matrix hemorrhage (GMH), intraventricular hemorrhage (IVH), intraparenchymal hemorrhage (IPH), and cerebellar hemorrhage represent the spectrum of ICHs in premature newborns [1]. These brain injuries can affect premature newborns’ morbidity, mortality, and neurodevelopment. Therefore, it is essential to identify ICH early and accurately in premature newborns to limit injury sequelae [2].

Approximately 600,000 children with head injuries visit emergency rooms annually [3]. ICH from other sources, including suspected nonaccidental trauma, is considered in many infants [3]. Radiation dangers are higher for children and newborns. There is one fatal malignancy per 1,000-5,000 head MRIs in children under five years of age [4]. Younger patients are more in danger because of quickly dividing tissues and a high number of remaining life years. Radiation exposure of youngsters should be kept as low as possible for safety [5].

The gold standard for examination of the ICH is magnetic resonance imaging (MRI). This is the most commonly employed method for the detection of ICH in premature infants because of its high sensitivity and specificity [6]. However, the use of cranial ultrasound for ICH diagnosis can be a more feasible option because of its portable characteristics, ionizing radiation-free nature, ease of handling and use, and ability to be performed safely more than once [7].

Squires et al. (2022) found ultrasonography had 100% sensitivity and 93.3% specificity in diagnosing grade III GMH using MRI but 22-50% sensitivity in diagnosing IPH, or IVH [8]. Guillot et al. (2020) reported cranial ultrasound’s sensitivity in detecting low-grade GMH and IVH was 77%, whereas its specificity was 79% [9]. Cranial ultrasound can help in the preoperative diagnosis of ICH, enabling the surgeon to anticipate and plan preoperatively. However, the existing literature is ambivalent about the diagnostic performance of cranial ultrasound, with sensitivity from as low as 77% to as high as 100% [8,9].

Ultrasound is a hardware- and operator-dependent modality. Therefore, determining its diagnostic accuracy in our setup with the available hardware and skills is essential. Moreover, the diagnostic performance depends upon the incidence of disease, which varies from 36% to 45% in extremely preterm infants having a birth weight of less than 750 grams [10]. The author could not find a single study in their radiology department or the Lahore region on this topic. Therefore, owing to the contrasting findings in the existing literature, the hardware- and operator-dependent features of ultrasound and the lack of any Lahore-based published literature necessitate this research to ensure evidence-based practice in the clinical radiological diagnostic domain. Moreover, cranial ultrasound will also provide advantages, such as its portability, real-time findings, cost-effectiveness, being ionizing radiation-free, and ability to repeat multiple times in cranial areas. These advantages make its use favorable if its validity is ensured [11]. The aim is to determine the diagnostic accuracy of cranial ultrasound for the identification of ICH in preterm neonates by taking MRI as the gold standard.

## Materials and methods

Study design, setting, and duration

A cross-sectional study was carried out to determine the diagnostic accuracy of ultrasonography. The research was conducted at the Department of Diagnostic Radiology, Children's Hospital Lahore, Pakistan. The duration of the study was six months. It was carried out from June 19, 2018 to December 18, 2018.

Sample size and sampling technique

The sample size of 103 cases was calculated using a 95% confidence interval with an expected sensitivity of 50.0% with a 13% margin of error, a specificity of 87.5% with a 12% margin of error, and a prevalence of disease of 39.0% [12]. A consecutive sampling (nonprobability) was used for patient selection.

Inclusion criteria

Neonates of both genders (neonates <28 days of age) were included in this study. Preterm neonates with suspected ICH who presented to the tertiary care hospital with any of three symptoms, e.g., altered state of consciousness (Modified Glasgow Coma Scale (NCS) < 9/15), weakness of any limb (muscle power < 4/5), or unequal pupils, were examined. Patients come with consent and are referred to the radiology department with ICH undergoing cranial ultrasound and later MRI.

Exclusion criteria

Patients with known congenital central nervous system (CNS) malformations and patients in whom ultrasound and MRI could not be performed within 48 hours of each other were excluded.

Data collection and diagnostic criteria of ICH

One hundred and three patients attending the neurology department at Children's Hospital Lahore who were referred to the radiology department were considered. They were included if the inclusion criteria were met, then they enrolled in this study. The patients enrolled after getting parental or guardian informed consent. Patients’ age and gender were noted. Neonates who were born before 37 weeks of gestation (determined by the last menstrual period) and presented with an altered state of consciousness (GCS < 9/15), weakness of any limb of the body (power of muscle < 4/5), or unequal pupils were labeled as suspected ICH. Cranial ultrasound was performed by skilled pediatric sonographers utilizing ultrasound (Zonare Medical Systems, Mountain View, USA). Curved and linear array transducers (8-17 MHz) were used to acquire consistent coronal and sagittal pictures via the anterior fontanelle. The transducer angle was adjusted to assess the cerebral peripheral. For each exam, penetration depth, ultrasound foci, ultrasound gain, and tissue brightness were fine-tuned. All these are standardized techniques to obtain images. MRI findings were considered the gold standard. The ultrasound results were evaluated for diagnostic accuracy (ultrasound can discriminate between preterm neonates with and without ICH), sensitivity (ultrasound can confirm ICH in preterm neonates), and specificity (ultrasound can rule out ICH in preterm neonates) using true positive, false positive, true negative, and false negative tests [13].

MRI and ultrasound procedure

An irregular hyperechoic mass on the ultrasound was labeled as ICH [14]. However, patients with an altered state of consciousness for the past 1 to 12 hours having an isointense to hypointense shadow on a T1-weighted MRI or a hyperintense image on a T2-weighted MRI were labeled as having acute ICH [15].

Patient’s demographic details, for example, age and gender, were noted and recorded into the self-designed pro forma along with ultrasound and MRI findings of ICH. All the ultrasound examinations were performed on the same machine by the same consultant radiologist (with eight years of teaching experience) to eliminate bias. Confounding variables have been controlled through exclusion.

Statistical analysis

All the collected data were entered and analyzed through IBM SPSS Statistics for Windows, Version 27 (Released 2020; IBM Corp., Armonk, New York, USA). Numerical variables and age have been presented by mean ± standard deviation. Categorical variables, that is, gender, ultrasound, and MRI findings of ICH, were present as frequency and percentage. The two-by-two (2 x 2) table has been generated to calculate the sensitivity, specificity, and positive and negative predictive values of ultrasound, taking MRI as the gold standard. The poststratification of the data has been carried out across age and gender to determine the role of effect modifiers. Moreover, the poststratification two-by-two table has been generated to calculate the sensitivity, specificity, and positive and negative predictive values of ultrasound, taking MRI as the gold standard.

Reporting guidelines and efforts to reduce bias

The Strengthening the Reporting of Observational Studies in Epidemiology (STROBE) Statement was followed in the writing of the manuscript [16]. Standardized techniques and established tools were utilized in this study to reduce the risk of bias. Moreover, the nonprobability consecutive sampling technique was utilized to prevent selection bias.

Ethical considerations

The study was carried out using the Declaration of Helinski ethical guidelines. Informed consent from attendant parents or guardians was also taken to ensure willingness. The patient attendant had the right to leave the study at any time. The anonymity and confidentiality of patients were maintained by de-identifying the data. The data will be published in a publicly available journal for the dissemination of findings for the benefit of patients in the future.

## Results

In this study, 103 neonates were included with a mean age of 9.9 ± 6.6 days (range of 1-27 days). The majority of the neonates (n = 71, 68.9%) were aged 1-14 days. There were 53 (51.5%) male and 50 (48.5%) female neonates (Table 1).

**Table 1:** Baseline characteristics of study sample (n = 103)

Baseline variables	Sample f (%)
Age (days) mean + SD	9.9 ± 6.6
1-14 days n (%)	71 (68.9%)
15-27 days n (%)	32 (31.1%)
Gender	
Male n (%)	53 (51.5%)
Female n (%)	50 (48.5%)

ICH was diagnosed in 45 (43.7%) neonates using cranial ultrasound and confirmed in 39 (37.9%) neonates using MRI. However, both modalities diagnosed approximately an equal number of cases as being without ICH: 58 (56.3%) and 64 (62.1%), respectively (Table 2).

**Table 2:** Diagnosis of intracranial hemorrhage on cranial ultrasound and MRI (n = 103)

Modality	Intracranial hemorrhage	Frequency (f)	Percent (%)
Cranial ultrasound	Yes	45	43.7%
	No	58	56.3%
	Total	103	100%
MRI	Yes	39	37.9%
	No	64	62.1%
	Total	103	100%

In Table 3, cranial ultrasound identified 39 out of 45 cases of ICH. However, MRI identified all 45 cases of ICH. There were 58 true negative cases, for which both modalities were agreed. There were no false negative cases reported by cranial ultrasound. The diagnostic performance of cranial ultrasound is comparable on the basis of these statistics. Therefore, the diagnostic performance of cranial ultrasound still showed significant performance which is confirmed by MRI.

**Table 3:** Diagnostic performance of cranial ultrasound in diagnosing intracranial hemorrhage (n = 103) ^a^True positive = 39; ^b^False negative = 0; ^c^False positive = 6; ^d^True negative = 58

Comparison of diagnostic performance using 2 x 2 contingency table		MRI		Total
		Intracranial hemorrhage	No	
Cranial ultrasound	Intracranial hemorrhage	39^a^	6^c^	45
	No	0^b^	58^d^	58
	Total	39	64	103

In Table 4, cranial ultrasound had 100.0% sensitivity, 90.62% specificity, 94.17% accuracy, 86.67% positive predictive value, and 100.0% negative predictive value for detecting ICH in preterm neonates with MRI as the gold standard. The sensitivity is 100% and the specificity is 90.62% with 94.17% accuracy.

**Table 4:** The detailed diagnostic performance parameters of cranial ultrasound ^a^True Positive = 39; ^b^False Negative = 0; ^c^False Positive = 6; ^d^True Negative = 58

Statistic	Formula	Value
Sensitivity	a / a + b	100.0%
Specificity	d / c + d	90.62%
Accuracy	a + d / a + b + c + d	94.17%
Disease prevalence	a + b / a + b + c + d	37.86%
Positive predictive value	a / a + c	86.67%
Negative predictive value	d / b + d	100.0%

The diagnostic performance was assessed by poststratification of data across age and gender. In the two age strata, there is no difference between sensitivity, but there is a fractional difference in terms of specificity among both strata showing fractionally better specificity among the 1-14 days age strata. In gender strata, there is no difference between male and female neonates to diagnose performance factors such as sensitivity, specificity, and positive and negative predicted values. Therefore, the diagnosis performance by age group and gender was persistent and showed consistency (Table 5).

**Table 5:** Diagnostic performance of cranial ultrasound in diagnosing intracranial hemorrhage across age group and gender (n = 103) SN: sensitivity, SP: specificity, AC: accuracy, PPV: positive predictive value, NPV: negative predictive value, PR: prevalence, ICH: intracranial hemorrhage

Age	Cranial ultrasound	MRI		Total	Diagnostic performance
		ICH	No		
1-14 days (n = 71)	ICH	27	4	31	SN = 100.0%, SP = 90.91%, PPV = 87.10%, NPV = 100.0% AC = 94.37%, PR = 38.03%
	No	0	40	40	
	Total	27	44	71	
15-27 days (n = 32)	ICH	12	2	14	SN = 100.0%, SP = 90.00%, PPV = 85.71%, NPV = 100.0%, AC = 93.75%, PR = 37.50%
	No	0	18	18	
	Total	12	20	32	
Male (n = 53)	ICH	20	3	23	SN = 100.0%, SP = 90.91%, PPV = 86.96%, NPV = 100.0%, AC = 94.34%, PR = 37.74%
	No	0	30	30	
	Total	20	33	53	
Female (n = 50)	ICH	19	3	22	SN = 100.0%, SP = 90.32%, PPV = 86.36%, NPV = 100.0% AC = 94.00%, PR = 38.00%
	No	0	28	28	
	Total	19	31	50	

## Discussion

Overall, the age of neonates ranged from 1 to 27 days, with a mean of 9.9 ± 6.6 days. Most neonates (n = 71, 68.9%) were 1-14 days old. There were 53 (51.5%) male and 50 (48.5%) female neonates. Neonatal ultrasound detected ICH in 45 (43.7%) neonates, and MRI detected it in 39 (37.9%). However, 58 (56.3%) and 64 (62.1%) of cases without cerebral bleeding are diagnosed by both methods. The cranial ultrasound had 100.0% sensitivity, 90.62% specificity, 94.17% accuracy, 86.67% positive predictive value, and 100.0% negative predictive value for detecting ICH in preterm neonates with MRI as the gold standard. ICH was diagnosed in 45 (43.7%) neonates on cranial ultrasound, whereas MRI confirmed ICH in 39 (37.9%) neonates. Poststratification across age and gender rated diagnostic performance approximately around with sensitivity as 100%, specificity as 90.62%, and accuracy as 94.17%. Diagnostic performance variables, including sensitivity, specificity, and positive and negative predicted values, are the same for male and female neonates. The diagnosing performance across age groups and genders was consistent and sustained.

In the present study, ICH was diagnosed in 37.9% of preterm neonates. A comparable frequency of 27.04% (20.9-32.3% at a 95% confidence interval) was reported in the low-resource center of Mizan Ethiopia using cranial ultrasonography. In another retrospective analysis, Wang et al. (2022) evaluated and compared the ICH-diagnosed preterm neonates using cranial ultrasound and MRI. The authors reported that 35.7% of ultrasound-diagnosed cases were confirmed by MRI, and their postnatal outcomes were compared, showing consistency in both imaging modalities [17]. Similarly, MacLeod et al. (2021) carried out a prospective cohort study and reported 34.2% of preterm neonates diagnosed with IVH using ultrasonography in Uganda [18]. Moreover, the 39% has been reported by Intrapiromkul et al. (2013) in the USA [12], whereas Radicioni et al. (2014) and Debbarma et al. (2017) reported it to be 32.7% in Italy [19] and 29.0% in India [20], respectively, using ultrasonography.

In the present study, cranial ultrasound was 100.0% sensitive, 90.6% specific, and 94.2% accurate in diagnosing ICH in preterm neonates. Our observation matches with that of Intrapiromkul et al. (2013), whose diagnostic performance statistics are consistent with this study, reporting cranial ultrasound to be 100.0% sensitive and 93.3% specific in the diagnosis of ICH in preterm neonates [12]. A similar specificity of 87.5% has also been reported by Elkhunovich et al. (2018); however, the sensitivity reported is lower at 50% [21]. Squires et al. (2022) compared ultrasound techniques to assess diagnostic accuracy in diagnosing ICH among preterm neonates. The diagnostic performance of cranial ultrasound showed a sensitivity of 93.3% and specificity of 100%, which somehow differs from the findings of this study. The authors also indicated that the sensitivity to diagnose subdural hemorrhage and IPH reduced to between 22.2% and 50% [8]. Therefore, there is variability in the diagnostic performance of cranial ultrasound, which emphasizes the need for further research in the future to validate the diagnostic performance findings to ensure evidence-based practice with a more robust study design. Conversely, Allen et al. (2023) carried out a systematic review and meta-analysis including 2,426 patients with a mean age of 60.1 years from 44 studies and somehow found a similar sensitivity, specificity, and accuracy of 88%, 81%, and 94%, respectively, for ICH patients. However, all these prognostic markers reduced the diagnosis of neurodegenerative disease using cranial ultrasound to 80%, 71%, and 76%, respectively. The overall diagnostic accuracy for cranial ultrasound in adults was also reported to be above 80% in this meta-analysis [22].

ICH was diagnosed in 45 (43.7%) neonates on cranial ultrasound, whereas MRI confirmed ICH in 39 (37.9%) neonates. Moreover, the increased specificity and sensitivity of cranial ultrasound are significant and improved, especially for ICH. Although cranial ultrasound is highly sensitive and specific, Ho et al. (2023) indicated that cranial ultrasound is not an alternative to MRI, particularly for small hemorrhages and white matter injury [23]. In this study, cranial ultrasonography showed significant diagnostic performance with high sensitivity and specificity for patients with ICH.

The poststratification of data across ages 1 to 27 days and equal distribution of gender among both modalities showed consistent results. Limited variability or difference in diagnostic statistics sensitivity, specificity, and accuracy was observed. Allen et al. (2023) also found there is no variability reported in the literature on the diagnostic performance of cranial ultrasonography [22].

Cranial ultrasonography continues to hold an important place in neonatal care. In skilled hands, neonatal brain sonography is invaluable. Allen et al. (2023) reported that most studies have not mentioned the sonographer’s specialization and academic background. The study also mentioned that similar studies did not use more than one sonographer or diagnostic image observer to utilize observer interrater agreement on the diagnostics of the images [22]. However, this study’s findings are interpreted by one sonography specialist from the past eight years in a neurology domain, which enhances the validity of the findings. Therefore, there is a need for future research involving more than one sonographer, which will help further validate the evidence. Lack of exposure has left many radiologists unfamiliar with newborn cranium imaging [22]. Using this approach involving more than one observer optimally can answer many clinical questions. The portability, lack of neonate transportation to radiology services, and quantity of diagnostic information of cranial ultrasound make this study notable.

Cranial sonography has the highest impact on neonates suspected to have meningitis and its complications; perinatal ischemia, particularly periventricular leukomalacia; and hydrocephalus resulting from a multitude of causes and hemorrhage [24]. In a premature infant, cerebral hemorrhage can be diagnosed using cranial ultrasound [25]. Although the gold standard for the examination of ICH is MRI, studies have shown cranial ultrasound to be a reliable bedside tool because of its noninvasiveness and ionizing radiation-free characteristics, which make it ideal and advocate for its preferred use in future practice [26]. However, the evidence of its diagnostic accuracy in such neonates was limited and variable [22], which necessitated carrying out the present study.

A limitation of this study was that ultrasound is a hardware- and operator-dependent tool and is frequently affected by interobserver variability. Only one observer (a sonographic specialist) is involved in this study. However, it is recommended in future studies to include more than one observer to add a more critical appraisal of data and the interpretation of images through an interobserver agreement approach. This is a single-center experience in central Punjab, Pakistan. There is a need for multicenter studies in this region while considering an interobserver agreement approach to establish the role of ultrasound with more certainty and critical appraisal. The power of a sample of the study is found with appropriate tests to ensure the study’s comprehensive power. Potential bias was reduced by utilizing STROBE Statement guidelines and standardized methods. More robust study designs, such as randomized controlled trials or longitudinal study designs, could be used in future studies to validate the findings.

## Conclusions

Cranial ultrasound was found to be 100.0% sensitive, 90.6% specific, and 94.2% accurate in diagnosing ICH in preterm neonates because of its noninvasiveness and ionizing radiation-free nature, and bedside availability advocates for cranial ultrasound as preferred for use in clinical practice. The observations were made by one sonographer, which is one of the limitations. This is the first study of this kind. The authors recommend future researchers conduct multicenter studies to increase the generalizability of the findings.
